# Anatomical connections among the depressor supercilii, levator labii superioris alaeque nasi, and inferior fibers of orbicularis oculi: Implications for variation in human facial expressions

**DOI:** 10.1371/journal.pone.0264148

**Published:** 2022-03-01

**Authors:** Mi-Sun Hur, Seunggyu Lee, Han-Sung Jung, Richard A. Schneider

**Affiliations:** 1 Department of Anatomy, Daegu Catholic University School of Medicine, Daegu, Korea; 2 Division of Applied Mathematical Sciences, Korea University, Sejong, Korea; 3 Division in Anatomy and Developmental Biology, Department of Oral Biology, Taste Research Center, Oral Science Research Center, BK21 FOUR Project, Yonsei University College of Dentistry, Seoul, South Korea; 4 Department of Orthopaedic Surgery, University of California at San Francisco, San Francisco, California, United States of America; Ohio State University, UNITED STATES

## Abstract

The aim of this study was to determine how the depressor supercilii (DS) connects to the levator labii superioris alaeque nasi (LLSAN) and inferior fibers of the orbicularis oculi (OOc INF) in the human midface. While grimacing, contraction of the DS with fibers connecting to the LLSAN and OOc INF can assist in pulling the medial eyebrow downward more than when these connecting fibers are not present. Contraction of these distinct connecting fibers between the DS and the LLSAN can also slightly elevate the nasal ala and upper lip. The DS was examined in 44 specimens of embalmed adult Korean cadavers. We found that the DS connected to the LLSAN or the OOc INF by muscle fibers or thin aponeuroses in 33 (75.0%) of the 44 specimens. The DS was connected to both the LLSAN and OOc INF by muscle fibers or aponeuroses and had no connection to either in 5 (11.4%) and 11 (25.0%) specimens, respectively. The DS was connected to the LLSAN by the muscle fibers and thin aponeuroses in 6 (13.6%) and 4 (9.1%) specimens, respectively. The DS was connected to the OOc INF by the muscle fibers and thin aponeuroses in 5 (11.4%) and 23 (52.3%) specimens, respectively. Our findings regarding the anatomical connections of the glabellar region DS to the midface LLSAN and OOc INF provide insights on the dynamic balance between the brow depressors such as the DS and brow-elevating muscle and contribute to understanding the anatomical origins of individual variation in facial expressions. These results can also improve the safety, predictability, and aesthetics of treatments for the glabellar region with botulinum toxin type A and can be helpful when performing electromyography.

## Introduction

**Fig 1:**
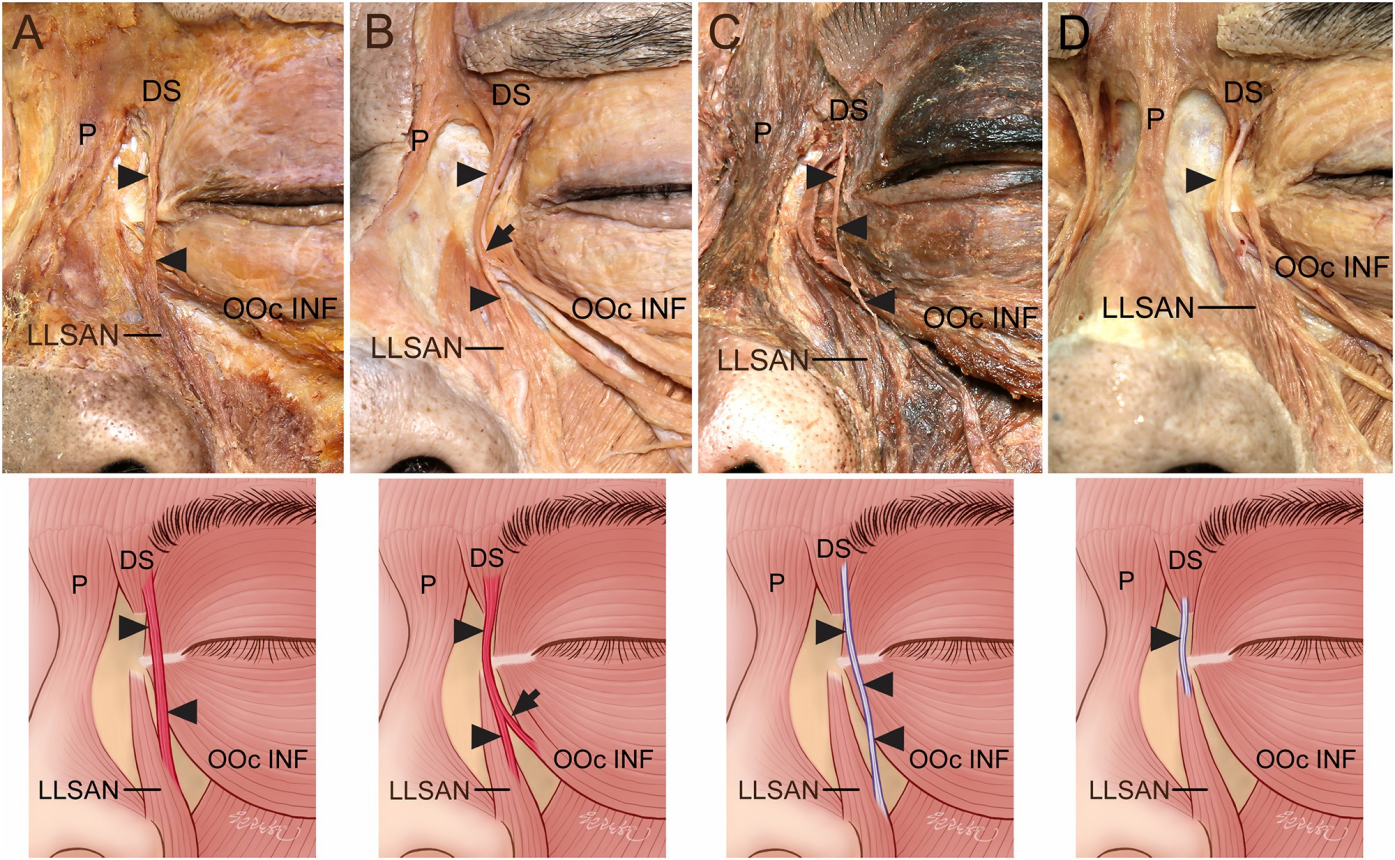
Connection of the DS to the LLSAN. (A) The DS was connected to the originating fibers of the LLSAN by the muscle fibers (arrowheads). (B) Some connecting muscle fibers (arrowheads) between the DS and LLSAN were connected with the OOc INF (arrows). (C) The DS was connected to the middle portion of the LLSAN by a thin aponeurosis (arrowheads). (D) The DS was connected to the originating fibers of the LLSAN by a thin aponeurosis (arrowheads).

The mimetic muscles are an assemblage of striated skeletal muscles that are innervated by the facial nerve (i.e., cranial nerve VII) and that control a broad range of voluntary and involuntary movements such as frowns and smiles, which are essential forms of non-verbal communication among humans. Charles Darwin eloquently argued in *The Expression of Emotion in Man and Animals* that the mimetic muscles convey intrinsic and universal emotions including sadness, anxiety, grief, joy, happiness, determination, anger, disgust, horror, pride, surprise, fear, and shame [1]. While grimacing or frowning, several facial muscles can be involved (Fig 1). The corrugator supercilii (CS) is a small pyramidal muscle located at the medial end of each eyebrow, lying deep to the frontalis and orbicularis oculi (OOc). The OOc is a broad, flat, elliptical muscle that surrounds the circumference of the orbit and is the sphincter muscle of the eyelids [2–4]. The depressor supercilii (DS) originates from the frontal process of the maxilla and inserts into the skin in the medial third of the eyebrow and into the OOc complex. The DS can be regarded as the fourth part of the OOc [5]. The procerus (P) is a small muscle that overlies the nasal bone. The frontalis of the occopitofrontalis arises from the epicranial aponeurosis and inserts into the skin of the eyebrow and the root of the nose. The levator labii superioris alaeque nasi (LLSAN) lies in the sulcus between the nose and cheek. The LLSAN arises from the upper part of the frontal process of the maxilla, and it descends to insert partly into the ala of the nose and partly into the skin of the lateral half of the upper lip. The LLSAN raises the lateral half of the upper lip and the wing of the nose [2–4].

Sir Charles Bell, who Darwin cited often, believed that one of the mimetic muscles, the CS, was “*the most remarkable muscle of the human face*. *It knits the eyebrows with a peculiar and energetic meaning*, *which unaccountably*, *but irresistibly*, *conveys the idea of mind and sentiment*” [6]. The CS is part of the glabellar complex, which also includes the DS, P, and the medial and superior fibers of the OOc [7]. These muscles contract in different directions to create dynamic wrinkles that appear and disappear rapidly while expressing emotions. Dynamic wrinkles become more permanent with age, and so for cosmetic purposes they can be treated temporarily using botulinum toxin (BoNT) type A to target the glabellar complex and improve moderate-to-severe frown lines (i.e., glabellar lines) between the eyebrows [8]. Normally, the dynamic balance of the eyebrows is maintained by the brow-elevating frontalis along with the brow depressors that include the CS, P, DS, and OOc [9]. DS contraction pulls the medial eyebrow down, creating a menacing expression. Contracting the CS pulls the eyebrow toward the middle and downward, creating vertical lines between the eyebrows. Contracting the P depresses the medial eyebrow, creating a horizontal wrinkle between the eyebrows. Contracting the orbital region of the OOc lowers and protrudes the eyebrow [10]. The force applied to the DS is directed toward its origin point on the orbital rim. Simulated DS contractions appear to act on the medial head of the eyebrow to depress the eyebrow more directly than the forces on the medial head of the orbital portion of the OOc [11]. BoNT type A use, however, can cause an imbalance between these facial muscles and may result in various subtle changes to facial expressions [8]. Whenever a muscle in a certain region is weakened, the force equilibrium shifts, and facial skin is pulled in the direction of the opposing muscles [12]. Different muscle patterns may also cause unexpected consequences from BoNT type A treatment [9]. Glabellar muscles are closely interconnected, and so BoNT type A injected into the targeted CS muscle will rapidly diffuse to the surrounding muscles [13]. Common adverse effects from treating glabellar wrinkles with BoNT type A include headaches, bruising, facial expression alteration, and ptosis [13]. Steinsapir et al. (2015) [14] therefore suggested a microdroplet BoNT type A injection method to selectively weaken the eyebrow depressors causing the brow to lift. A key muscle to understand in this context is the DS.

Several authors have confirmed the distinction of the DS [11, 15, 16], although there has been some confusion regarding whether the DS is part of the OOc or the CS [15, 17, 18]. Daniel and Landon (1997) [15] indicated that the DS is clearly separated from both the OOc and CS by distinct anatomical planes, each containing neurovascular structures and adipose tissue. Those authors also reported that the bone origin is discrete, the vertical muscle belly is easily isolated, and the medial eyebrow insertion is clearly visible. Cook et al. (2001) [16] suggested that the origin, insertion, and anatomy of the DS creates a depressing action of the eyebrow, and the DS has a distinct origin and insertion, as observed histologically. If BoNT type A treatment paralyzes the DS, the eyebrows may lift upward [13], indicating that the DS can act independently. Waller et al. (2006) [19] suggested that glabellar wrinkles are more likely to be caused by contraction of the DS than the CS. Therefore, considering the individual effects of the DS on eyebrow movements and the glabellar skin is critical to achieve a better understanding of the interactions among the DS, other glabellar muscles, and the frontalis. Accurate knowledge of DS anatomy can help elucidate the origins of variations in human facial expressions and can reduce unexpected alterations of the position and shape of the medial eyebrow, as well as the risk of facial asymmetry from BoNT type A injections.

Prior observations on the different contraction directions for each glabellar muscle and a microdroplet BoNT type A injection method for selective glabellar muscles indicate that precise anatomical data on the glabellar muscles are required for the delicate and accurate use of BoNT type A injections as a treatment. The connections among the DS and the LLSAN or inferior fibers of the OOc (OOc INF) have not been reported previously. Moreover, the extent to which these connections occur via muscle fibers or aponeuroses remain unclear, and this is important since the type of connection likely affects the movements of glabellar muscles as well as those in surrounding regions, and presumably can establish the range of motion during coordinated facial expression.

The aim of this study was to determine the anatomical connections of the glabellar DS to the midface LLSAN and OOc INF, as a means to identify individual variations in the topographic relationships that may influence facial expressions. This information will also assist in designing precise therapies for the glabellar region that involve BoNT type A, performing various types of facial surgeries, and in studies that use electromyography (EMG).

## Materials and methods

### Specimens and dissection

This study examined the DS in 44 specimens of embalmed adult Korean cadavers (10 males, 12 females; mean age of 72.1 years, age range of 40–94 years). All cadavers had been legally donated to the Catholic Kwandong University College of Medicine. This study was conducted in accordance with the Declaration of Helsinki. No transplant donor was from a vulnerable population, and all donors or their next of kin voluntarily provided written informed consent. This study was approved by the Institutional Review Board of the Catholic Kwandong University (IRB No. CKU-21-01-0101).

The upper and middle portions of the face were dissected to expose the DS, LLSAN, OOc, and their surrounding structures. A detailed dissection that focused on the anatomical connections of the DS to the LLSAN and OOc was performed. When the structures connecting the DS to the LLSAN and OOc were identified, they were followed to observe their courses, connection, and attachments.

## Results

The DS was connected to the LLSAN or the OOc INF by muscle fibers or aponeuroses in 33 of the 44 (75.0%) specimens. The DS was connected to both the LLSAN and OOc INF by muscle fibers or aponeuroses and had no connection to either in 5 (11.4%) and 11 (25.0%) specimens, respectively.

The DS was connected to the LLSAN by muscle fibers and thin aponeuroses in 6 (13.6%) and 4 (9.1%) of the 44 specimens, respectively (Fig 1). The muscle fibers or aponeuroses connecting to the DS and LLSAN were often attached to the originating fibers of the LLSAN and sometimes to the middle portion of the LLSAN. The connecting muscle fibers or aponeurosis between the DS and LLSAN tended to be thicker than those between the DS and OOc INF.

**Fig 2:**
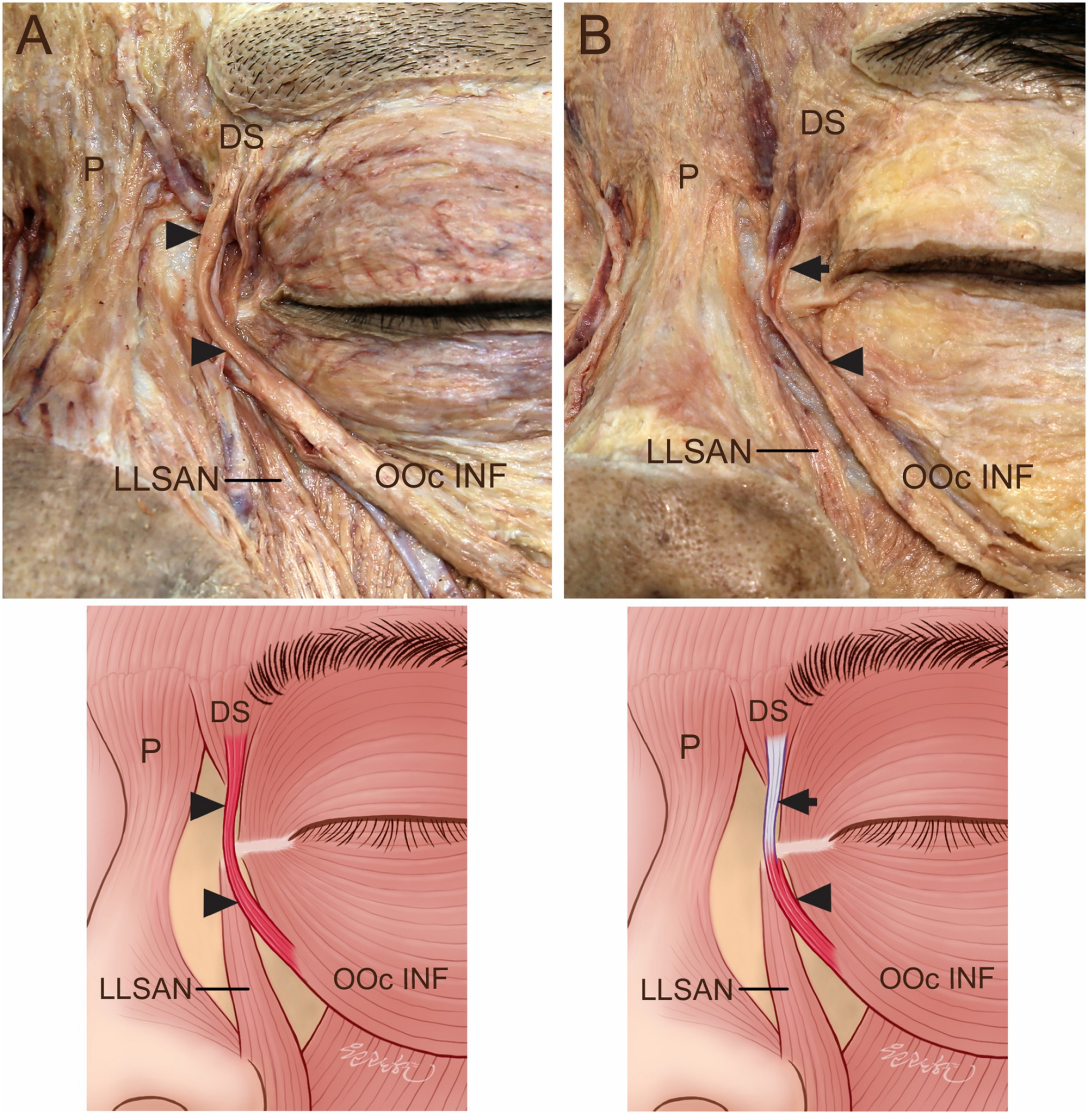
Connection of the DS to the OOc INF. (A) The DS was connected to the OOc INF by the muscle fibers (arrowheads). (B) The DS was connected to the OOc INF by the thin aponeurosis (arrows). Some muscle fibers (arrowheads) of the OOc INF were connected to the thin aponeurosis (arrows) coursing on the medial palpebral ligament, and the thin aponeurosis (arrows) of the OOc INF was connected to the DS.

The DS was connected to the OOc INF by muscle fibers and thin aponeuroses in 5 (11.4%) and 23 (52.3%) of the 44 specimens, respectively (Fig 2). In the cases where the DS was connected to the OOc INF by thin aponeuroses, the muscle fibers of the OOc INF replaced the thin aponeurosis, coursing on the medial palpebral ligament, and the thin aponeurosis of the OOc INF was then connected to the DS. One specimen had two thin aponeuroses between the DS and OOc INF.

**Fig 3:**
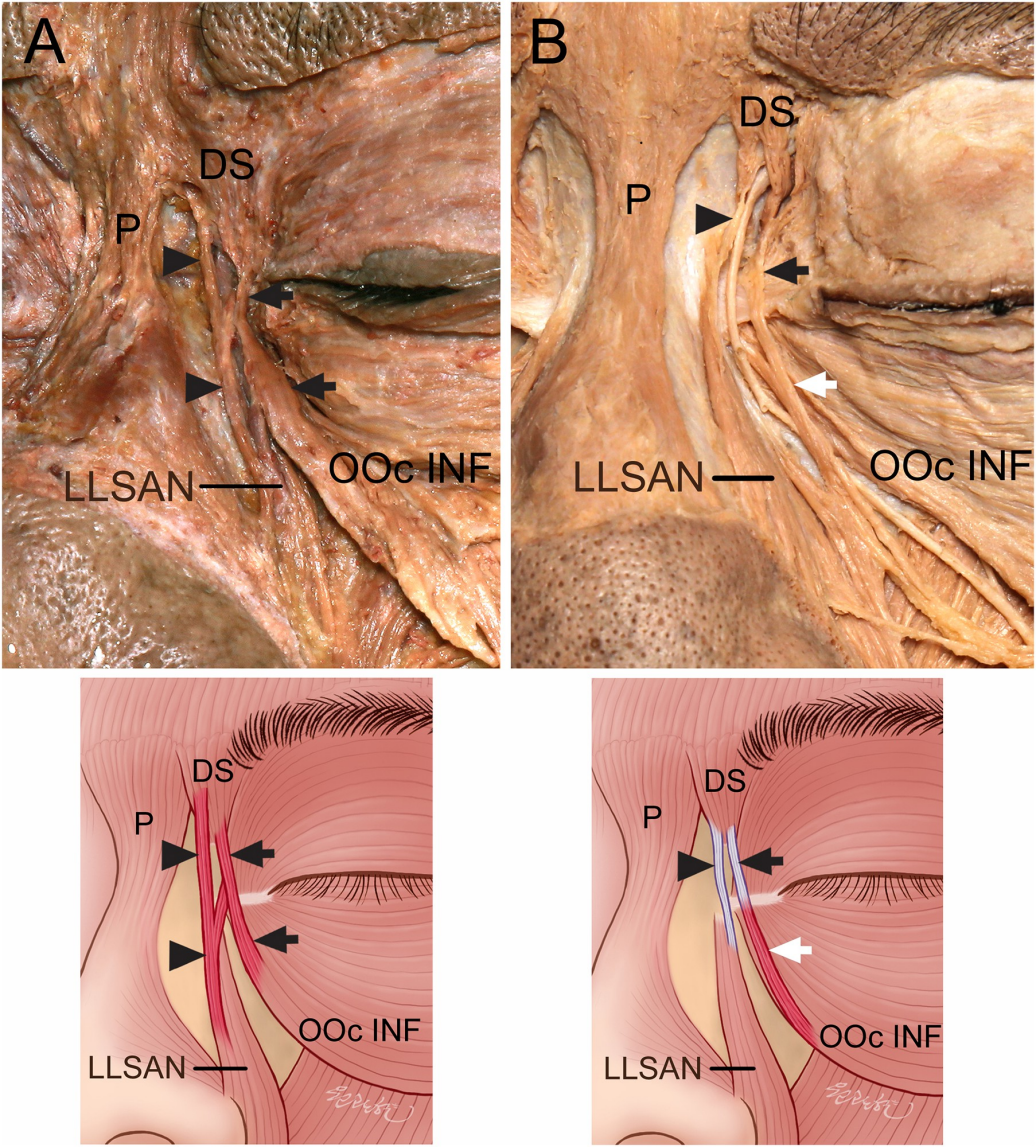
Connections of the DS to both the LLSAN and OOc INF. (A) Connecting muscle fibers were found between the DS and LLSAN (arrowheads) and between the DS and OOc INF (arrows). (B) Connecting aponeuroses were found between the DS and LLSAN (arrowheads) and between the DS and OOc INF (arrows). Some muscle fibers (white arrows) of the OOc INF were connected to the thin aponeurosis (black arrows) coursing on the medial palpebral ligament, and the thin aponeurosis (black arrows) of the OOc INF was connected to the DS.

Among the five specimens where the DS was connected to both the LLSAN and OOc INF, the DS of one specimen had muscle fibers connecting to the LLSAN and OOc INF. The DS in one specimen had aponeuroses connected to the LLSAN and OOc INF. The DS in three specimens had the muscle fibers and aponeurosis connected to the LLSAN and OOc INF, respectively. In the specimen that had muscle fibers of the DS connected to both the LLSAN and OOc INF, two muscle fibers were connected before combining with the DS (Fig 3).

The muscle fibers or a thin aponeurosis connected to the DS and LLSAN were often located slightly medial to the angular vein, and sometimes slightly lateral or superficial to the angular vein, whereas the muscle fibers or a thin aponeurosis connected to the DS and OOc INF were often located slightly lateral to and sometimes superficial to the angular vein. Two aponeuroses were found between the DS and OOc INF in one specimen, with each aponeurosis located just medial and lateral to the angular vein.

Sex-related differences in the prevalence of the muscle fibers or the aponeurosis connecting the DS and LLSAN or between the DS and OOc INF were analyzed. Muscle fibers or the aponeurosis were connected to the DS and LLSAN and the DS and OOc in 5 males and 5 females, and in 15 males and 13 females, respectively. However, our analysis indicated a significant difference between males and females in the prevalence of muscle fibers connecting to the DS and LLSAN or the DS and OOc INF: all five specimens with muscle fibers connected to the DS and OOc INF were male, while males constituted four of the six specimens with muscle fibers connected to the DS and LLSAN. The presence of muscle fibers or aponeurosis connecting to the DS and LLSAN and to the DS and OOc was found to be symmetrical in one male and two females, and in six males and four females, respectively.

## Discussion

This study found that the glabellar muscle DS was most often connected to muscle fibers or the aponeurosis by the LLSAN or OOc INF, accounting for 75.0% of the specimens. Hur (2017) [20] reported connections between the P and the LLSAN or transverse nasalis, indicating that the action of the P is closely related to movements in the nasal area. The DS and the P that constitute the glabellar muscles were therefore thought to have close anatomical and functional relationships with the middle facial muscles. The connecting muscle fibers or aponeurosis linking the glabellar region to the midface may affect facial expressions and may cause unexpected outcomes of BoNT type A treatment and EMG analysis.

**Fig 4:**
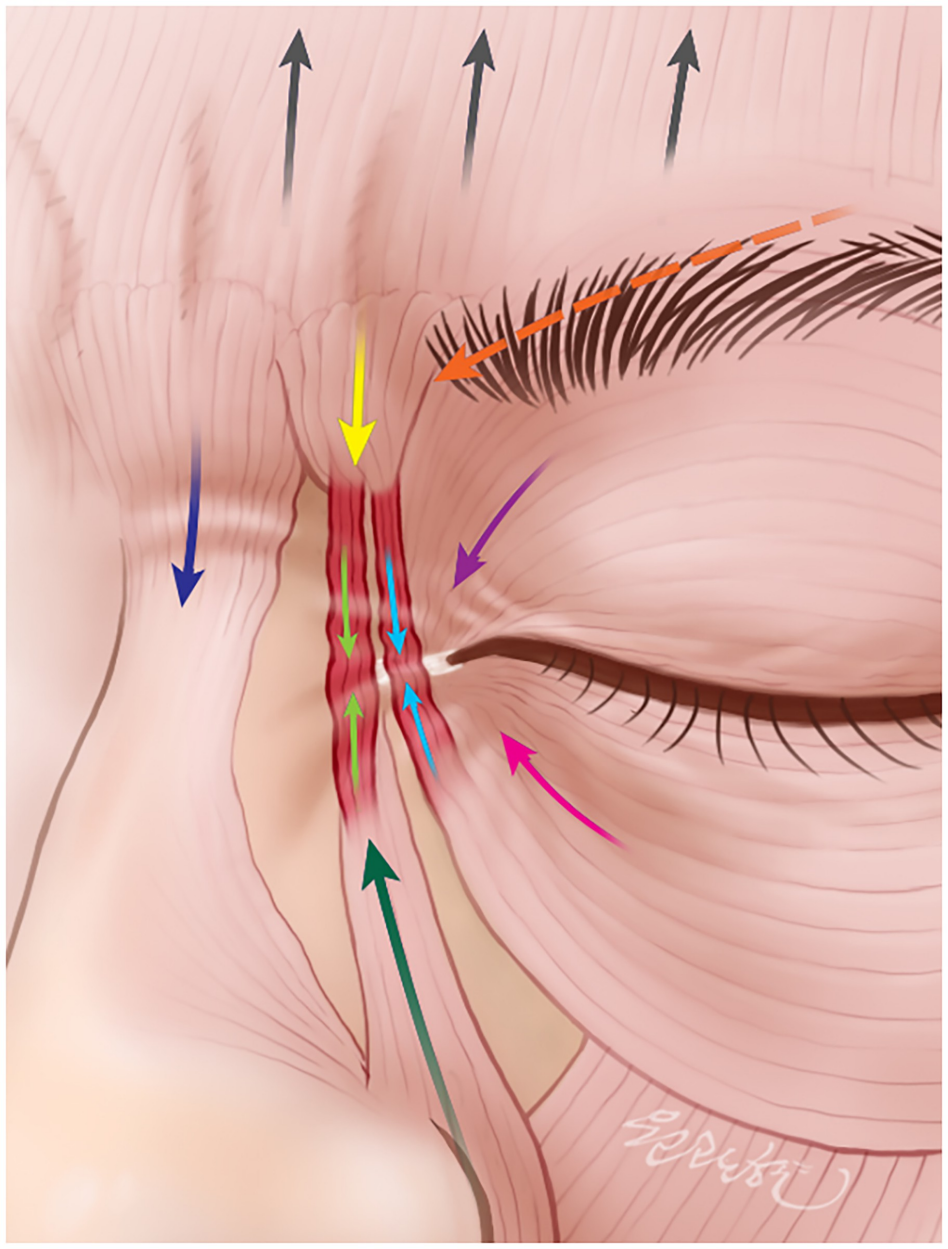
Contractions and vectors of the glabellar muscles and the fibers connecting the DS and the LLSAN and OOc INF while grimacing. Contracting the DS (yellow arrow) will pull the medial eyebrow down. Contracting the CS (orange arrow) pulls the eyebrow medially and down. Contracting the P (dark-blue arrow) depresses the medial eyebrow. Contracting the superior fibers of OOc (purple arrow) lowers and protrudes the eyebrow inferomedially. Contracting the OOc INF (pink arrow) elevates the cheek superomedially. Contracting the frontalis (black arrows) elevates the eyebrow. Contracting the LLSAN (green arrow) elevates the nasal ala and upper lip. The fibers (yellow and green arrows) connecting the DS and LLSAN (green arrow) and those (light-blue arrows) connecting the DS and OOc INF (pink arrow) may assist in depressing the medial eyebrow, elevating the nasal ala, upper lip, and cheek.

During grimacing, the medial eyebrows depress, and the nasal ala and upper lip elevate. The associated contraction of the DS alongside its distinct fibers connecting to the LLSAN and OOc INF can assist in further pulling the medial eyebrow downward compared to when these connecting fibers are not present. Contraction of the fibers connecting the DS and LLSAN can also slightly elevate the nasal ala and upper lip (Fig 4). The connecting aponeurosis may detect and diffuse tension between the DS and LLSAN or between the DS and OOc INF so as to alter facial expressions.

The DS was connected to the LLSAN by muscle fibers or aponeuroses in 22.7% of the specimens examined. The LLSAN primarily has a mimetic function [21], being somewhat inactive during respiration but having a high level of activity during complex mimetic activities. The LLSAN controls various motions during forehead frowning, whistling, elevation of the upper lip and nasal ala, upper lip eversion, lip compression, blowing with cheek distension, nostril dilation, and other facial expressions [22]. We therefore suggest that the muscle fibers or aponeurosis connecting the DS to the LLSAN can assist in connecting glabellar and midface movements during these expressions.

Waller et al. (2006) [19] indicated that intramuscular electrical stimulation of the LLSAN lowered and protruded the glabellar region. It is possible that the fibers connecting the DS and LLSAN explain the medial eyebrow depression found in EMG investigations. Hur (2017) [20] reported that some medial fibers originating in the LLSAN superomedially extended to combine the area between the P and the DS or into the P in 21.2% of the specimens. In our study, some muscle fibers or aponeuroses of the LLSAN were connected to the DS in 22.7% of the specimens, which likely further pulls the DS downward.

The DS was connected to the OOc INF by muscle fibers or aponeuroses in 63.6% of the specimens. Between the DS and OOc INF, the aponeurosis was observed as a connection more often than the muscle fibers, whereas the muscle fibers were observed as a connection more often than the aponeurosis between the DS and LLSAN. The OOc plays an important role in facial expression and various ocular reflexes [2]. The lower half of the orbital portion of the OOc pulls the cheek skin and lower eyelids upward, causing wrinkles that radiate from the corner of the eye [3, 23]. Waller et al. (2006) [19] indicated that intramuscular electrical stimulation of the orbital portion of the OOc pushed the skin that is inferior and lateral to the eye medially and superiorly, causing bags and wrinkles in the skin below the eye. The OOc is a phasic mimetic muscle, and the marked predominance of type II muscle fibers within the OOc indicates its phasic protective function during rapid eye closure [24]. These muscle fibers or aponeurosis connecting the DS to the OOc INF may therefore assist in pulling the medial eyebrow and elevating the inferomedial orbital region for rapid movements of the orbital and glabellar regions simultaneously.

The variation among the specimens we examined both in the anatomy of the DS, LLSAN, OOc INF and in the tissues (i.e., muscle fibers versus aponeuroses) that interconnect these muscles is striking and points to underlying differences in the developmental programs that pattern the facial musculature. During embryonic development, the presumptive craniofacial muscles are surrounded by neural crest mesenchyme (NCM), which migrates into the facial primordia and produces numerous cell types including all the chondrocytes that make cartilage, osteoblasts that make bone, tenocytes that make tendons and aponeuroses, and ligamentous fibroblasts that make other muscle connective tissues [25–36]. In contrast, craniofacial muscles and all their skeletal muscle fibers are derived from paraxial mesoderm that migrates as myogenic precursors alongside NCM on route to the facial primordia [37–43]. Numerous aspects of craniofacial muscle pattern are regulated by NCM-derived connective tissues including fiber type, muscle orientation, and the precise locations of attachments [44–51]. This is like what happens in the limb where connective tissue fibroblasts provide essential signals that presage muscle pattern and guide both fast- and slow-twitch muscle differentiation [52–54]. The interactions between NCM and mesodermal mesenchyme not only ensure the structural integration necessary for attaining proper muscle function during development, but they also appear to help orchestrate the co-evolution of the musculoskeletal system across species [55, 56]. Such conclusions are based on transplant experiments between quail and duck embryos, which demonstrate that NCM-derived tendon and muscle connective tissues and the molecular signals that emanate from them, determine the species-specific shape of muscles, location of attachment sites, and local mechanical force environment [57–59]. Thus, in the case of the muscles of expression, NCM-mediated changes to associated connective tissues during development likely underlie the individual anatomical variation that we have observed in our study, could have functional implications among individuals and related species, and may reflect differences in an adaptive landscape for the musculoskeletal system that has influenced the course of human evolution.

The DS is common in chimpanzees, gorillas, and humans, and hence has been retained during the evolutionary history of hominids. The DS controls the supraorbital tactile vibrissae of lower primates. The glabellar and supraorbital musculature were found during a progressive stage of human evolution. Although the DS behaves somewhat similarly to the primitive muscle complexes of chimpanzees and gorillas, its occurrence increases in progressive cases, especially for Caucasians [60]. Waller et al. (2006) [19] reported that the upper face of humans appears more specialized for eyebrow movement than in the chimpanzee perhaps due to the increased signal value of the eyebrows. Likewise, as a product of domestication, dogs have achieved an ability to raise their eyebrows in a manner that seemingly solicits a nurturing response in humans (i.e., “puppy dog eyes”) by using the levator anguli oculi medialis muscle, which is not present in wolves [61].

The DS was observed in all the human specimens in the present study, and the muscle fibers or aponeuroses were often connected to the LLAN or OOc INF, which implies their roles in synergic actions that connect the movements of the glabellar and midface regions of humans. In contrast, the DS was present in only 50% of rhesus macaque *(Macaca mulatta)* specimens and was located between the P and LLSAN in *Hypolepis muelleri* specimens [62, 63]. The DS of chimpanzees (*Pan troglodytes*) is attached inferiorly to the skin of the nasal bone, and deep relative to the P [64], whereas the DS of humans is attached to the frontal process of the maxilla, lateral to the P.

Our findings regarding the anatomical connections of the glabellar region DS to the LLSAN and OOc INF in the midface contribute to understanding the dynamic balance between the brow depressors, such as the DS and the brow-elevating muscles. The ability to control eyebrow movements is a vital component of human social interactions that are facilitated by facial expressions [65–67] and our results help explain the anatomical basis for individual variation in this ability. Our results may also improve the safety, predictability, and aesthetics of the glabellar region during treatment involving BoNT type A and can help inform studies involving EMG.

## References

[1] Darwin C. The expression of the emotions in man and animals. London: J. Murray; 1872.

[2] Standring S. Gray’s Anatomy: The Anatomical Basis of Clinical Practice. 42nd ed. New York: Elsevier; 2020.

[3] Morris H. Morris’ Human Anatomy: A Complete Systematic Treatise. 10th ed. Philadelphia: Blakiston Co.; 1947.

[4] Woodburne RT, Burkel WE. Essentials of Human Anatomy. 9th ed. New York: Oxford University Press, 1994;246.

[5] Benedetto AV. Botulinum toxins in clinical aesthetic practice. 3rd ed. New York: CRC Press; 2018.

[6] Bell C. Essays on the anatomy and philosophy of expression. 2nd ed. London: John Murray; 1824.

[7] Flynn TC. Periocular botulinum toxin. Clin Dermatol. 2003; 21(6):498–504. doi: 10.1016/j.clindermatol.2003.11.015 . 14759583

[8] Kim HJ, Seo KK, Lee HK, Kim J. Clinical Anatomy of the Face for Filler and Botulinum Toxin Injection. Singapore: Springer; 2016.

[9] Pinar Y, Govsa F, Ozer MA, Ertam I. Anatomocosmetic implication rules of the corrugator supercilii muscle for youthful eye appearance. Surg Radiol Anat. 2016; 38(9):1045–1051. doi: 10.1007/s00276-016-1666-1 . 27021220

[10] De Maio M. Rzany B. Botulinum toxin in aesthetic medicine. New York: Springer; 2007. 10.1016/j.jaad.2006.12.038 17504724

[11] Knize DM. Muscles that act on glabellar skin: a closer look. Plast Reconstr Surg. 2000; 105(1):350–361. doi: 10.1097/00006534-200001000-00056 . 10627005

[12] Lorenc ZP, Smith S, Nestor M, Nelson D, Moradi A. Understanding the functional anatomy of the frontalis and glabellar complex for optimal aesthetic botulinum toxin type A therapy. Aesthetic Plast Surg. 2013; 37(5):975–983. doi: 10.1007/s00266-013-0178-1 . 23846022

[13] Seo KK. Botulinum Toxin for Asians. Singapore: Springer; 2017.

[14] Steinsapir KD, Rootman D, Wulc A, Hwang C. Cosmetic Microdroplet Botulinum Toxin A Forehead Lift: A New Treatment Paradigm. Ophthalmic Plast Reconstr Surg. 2015; 31(4):263–268. doi: 10.1097/IOP.0000000000000282 . 25216199

[15] Daniel RK, Landon B. Endoscopic forehead lift: anatomic basis. Aesthet Surg J. 1997; 17(2):97–104. doi: 10.1016/s1090-820x(97)80070-2 . 19327696

[16] Cook BE Jr, Lucarelli MJ, Lemke BN. Depressor supercilii muscle: anatomy, histology, and cosmetic implications. Ophthalmic Plast Reconstr Surg. 2001; 17(6):404–411. doi: 10.1097/00002341-200111000-00004 . 11766019

[17] Abramo AC. Anatomy of the forehead muscles: the basis for the videoendoscopic approach in forehead rhytidoplasty. Plast Reconstr Surg. 1995; 95(7):1170–1177. doi: 10.1097/00006534-199506000-00005 . 7761503

[18] Flowers RS. The open approach to forehead and brow lifting. Aesthet Surg J. 1998; 18(6):463–464. doi: 10.1016/s1090-820x(98)70082-2 . 19328180

[19] Waller BM, Vick SJ, Parr LA, Bard KA, Pasqualini MC, Gothard KM, et al. Intramuscular electrical stimulation of facial muscles in humans and chimpanzees: Duchenne revisited and extended. Emotion. 2006; 6(3):367–382. doi: 10.1037/1528-3542.6.3.367 . 16938079 PMC2826128

[20] Hur MS. Anatomical relationships of the procerus with the nasal ala and the nasal muscles: transverse part of the nasalis and levator labii superioris alaeque nasi. Surg Radiol Anat. 2017; 39(8):865–869. doi: 10.1007/s00276-017-1817-z . 28132092

[21] Bruintjes TD, Olphen AF, Hillen B, Weijs WA. Electromyography of the human nasal muscles. Eur Arch Otorhinolaryngol. 1996;253(8):464–469. doi: 10.1007/BF00179951 . 8950546

[22] Vitti M, Fortinguerra CR, Corrêa AC, König B Jr, Bérzin F. Electromyographic behavior of the levator labii superioris alaeque nasi. Electromyogr Clin Neurophysiol. 1974; 14(1):37–43. . 4457322

[23] Hollinshead WH. Anatomy for surgeons: Volume 1, the head and neck. 3rd ed. New York: Harper & Row; 1982.

[24] Happak W, Burggasser G, Gruber H. Histochemical characteristics of human mimic muscles. J Neurol Sci. 1988; 83(1):25–35. doi: 10.1016/0022-510x(88)90017-2 . 2964514

[25] Köntges G, Lumsden A. Rhombencephalic neural crest segmentation is preserved throughout craniofacial ontogeny. Development. 1996; 122(10):3229–3242. doi: 10.1242/dev.122.10.3229 . 8898235

[26] Le Lièvre CS, Le Douarin NM. Mesenchymal derivatives of the neural crest: analysis of chimaeric quail and chick embryos. J Embryol Exp Morphol. 1975; 34(1):125–154. 1185098

[27] Dupin E, Calloni GW, Le Douarin NM. The cephalic neural crest of amniote vertebrates is composed of a large majority of precursors endowed with neural, melanocytic, chondrogenic and osteogenic potentialities. Cell Cycle. 2010; 9(2):238–249. doi: 10.4161/cc.9.2.10491 . 20037475

[28] Le Lièvre CS. Participation of neural crest-derived cells in the genesis of the skull in birds. J Embryol Exp Morphol. 1978; 47:17–37. 722230

[29] Noden DM. The control of avian cephalic neural crest cytodifferentiation. I. Skeletal and connective tissues. Dev Biol. 1978; 67(2):296–312. doi: 10.1016/0012-1606(78)90201-4 . 738529

[30] Couly GF, Coltey PM, Le Douarin NM. The triple origin of skull in higher vertebrates: a study in quail-chick chimeras. Development. 1993; 117(2):409–429. doi: 10.1242/dev.117.2.409 . 8330517

[31] Noden DM, Schneider RA. Neural crest cells and the community of plan for craniofacial development: historical debates and current perspectives. Adv Exp Med Biol. 2006; 589:1–23. doi: 10.1007/978-0-387-46954-6_1 . 17076272

[32] Jheon AH, Schneider RA. The cells that fill the bill: neural crest and the evolution of craniofacial development. J Dent Res. 2009; 88(1):12–21. doi: 10.1177/0022034508327757 . 19131312 PMC3317957

[33] Jiang X, Iseki S, Maxson RE, Sucov HM, Morriss-Kay GM. Tissue origins and interactions in the mammalian skull vault. Dev Biol. 2002; 241(1):106–116. doi: 10.1006/dbio.2001.0487 . 11784098

[34] McBratney-Owen B, Iseki S, Bamforth SD, Olsen BR, Morriss-Kay GM. Development and tissue origins of the mammalian cranial base. Dev Biol. 2008; 322(1):121–132. doi: 10.1016/j.ydbio.2008.07.016 . 18680740 PMC2847450

[35] Morriss-Kay GM. Derivation of the mammalian skull vault. J Anat. 2001; 199(Pt 1–2): 143–151. doi: 10.1046/j.1469-7580.2001.19910143.x . 11523816 PMC1594961

[36] Yoshida T, Vivatbutsiri P, Morriss-Kay G, Saga Y, Iseki S. Cell lineage in mammalian craniofacial mesenchyme. Mech Dev. 2008; 125(9–10): 797–808. doi: 10.1016/j.mod.2008.06.007 . 18617001

[37] Noden DM. The embryonic origins of avian cephalic and cervical muscles and associated connective tissues. Am J Anat. 1983; 168(3):257–276. doi: 10.1002/aja.1001680302 . 6650439

[38] Noden DM. Patterning of avian craniofacial muscles. Dev Biol. 1986; 116(2):347–356. doi: 10.1016/0012-1606(86)90138-7 . 3732610

[39] Noden DM, Trainor PA. Relations and interactions between cranial mesoderm and neural crest populations. J Anat. 2005; 207(5): 575–601. doi: 10.1111/j.1469-7580.2005.00473.x . 16313393 PMC1571569

[40] Trainor PA, Tam PP. Cranial paraxial mesoderm and neural crest cells of the mouse embryo: co-distribution in the craniofacial mesenchyme but distinct segregation in branchial arches. Development. 1995; 121(8): 2569–2582. doi: 10.1242/dev.121.8.2569 . 7671820

[41] Trainor PA, Tan SS, Tam PP. Cranial paraxial mesoderm: regionalisation of cell fate and impact on craniofacial development in mouse embryos. Development. 1994; 120(9):2397–2408. doi: 10.1242/dev.120.9.2397 . 7956820

[42] Evans DJ, Noden DM. Spatial relations between avian craniofacial neural crest and paraxial mesoderm cells. Dev Dyn. 2006; 235(5):1310–1325. doi: 10.1002/dvdy.20663 . 16395689

[43] Couly GF, Coltey PM, Le Douarin NM. The developmental fate of the cephalic mesoderm in quail-chick chimeras. Development. 1992; 114(1):1–15. doi: 10.1242/dev.114.1.1 . 1576952

[44] Noden DM. The Role of the Neural Crest in Patterning of Avian Cranial Skeletal, Connective, and Muscle Tissues. Dev Biol. 1983; 96(1):144–165. doi: 10.1016/0012-1606(83)90318-4 . 6825950

[45] Noden DM, Marcucio R, Borycki AG, Emerson CP Jr. Differentiation of avian craniofacial muscles: I. Patterns of early regulatory gene expression and myosin heavy chain synthesis. Dev Dyn. 1999; 216(2):96–112. . 10536051 10.1002/(SICI)1097-0177(199910)216:2<96::AID-DVDY2>3.0.CO;2-6

[46] Marcucio RS, Noden DM. Myotube heterogeneity in developing chick craniofacial skeletal muscles. Dev Dyn. 1999; 214(3):178–194. . 10090145 10.1002/(SICI)1097-0177(199903)214:3<178::AID-AJA2>3.0.CO;2-4

[47] Noden DM, Francis-West P. The differentiation and morphogenesis of craniofacial muscles. Dev Dyn. 2006; 235(5):1194–1218. doi: 10.1002/dvdy.20697 . 16502415

[48] Sambasivan R, Kuratani S, Tajbakhsh S. An eye on the head: the development and evolution of craniofacial muscles. Development. 2011; 138(12): 2401–2415. doi: 10.1242/dev.040972 . 21610022

[49] Rinon A, Lazar S, Marshall H, Buchmann-Moller S, Neufeld A, Elhanany-Tamir H, et al. Cranial neural crest cells regulate head muscle patterning and differentiation during vertebrate embryogenesis. Development. 2007; 134(17):3065–3075. doi: 10.1242/dev.002501 . 17652354

[50] Grenier J, Teillet MA, Grifone R, Kelly RG, Duprez D. Relationship between neural crest cells and cranial mesoderm during head muscle development. PLoS One. 2009; 4(2):e4381. doi: 10.1371/journal.pone.0004381 19198652 PMC2634972

[51] Tzahor E. Heart and craniofacial muscle development: a new developmental theme of distinct myogenic fields. Dev Biol. 2009; 327(2):273–279. doi: 10.1016/j.ydbio.2008.12.035 . 19162003

[52] Kardon G. Muscle and tendon morphogenesis in the avian hind limb. Development. 1998; 125(20):4019–4032. doi: 10.1242/dev.125.20.4019 . 9735363

[53] Kardon G, Harfe BD, Tabin CJ. A Tcf4-positive mesodermal population provides a prepattern for vertebrate limb muscle patterning. Dev Cell. 2003; 5(6):937–944. doi: 10.1016/s1534-5807(03)00360-5 . 14667415

[54] Mathew SJ, Hansen JM, Merrell AJ, Murphy MM, Lawson JA, Hutcheson DA, et al. Connective tissue fibroblasts and Tcf4 regulate myogenesis. Development. 2011; 138(2):371–384. doi: 10.1242/dev.057463 . 21177349 PMC3005608

[55] Schneider RA. Neural crest and the origin of species-specific pattern. Genesis. 2018; 56(6–7):e23219. doi: 10.1002/dvg.23219 . 30134069 PMC6108449

[56] Woronowicz KC, Schneider RA. Molecular and cellular mechanisms underlying the evolution of form and function in the amniote jaw. Evodevo. 2019; 10(1):17. doi: 10.1186/s13227-019-0131-8 . 31417668 PMC6691539

[57] Tokita M, Schneider RA. Developmental origins of species-specific muscle pattern. Dev Biol. 2009; 331(2):311–325. doi: 10.1016/j.ydbio.2009.05.548 . 19450573 PMC2726847

[58] Solem RC. Eames BF, Tokita M, Schneider RA. Mesenchymal and mechanical mechanisms of secondary cartilage induction. Dev Biol. 2011; 356(1):28–39. doi: 10.1016/j.ydbio.2011.05.003 . 21600197 PMC3130809

[59] Woronowicz KC, Gline SE, Herfat ST, Fields AJ, Schneider RA. FGF and TGFbeta signaling link form and function during jaw development and evolution. Dev Biol. 2018; 444 Suppl 1:S219–S236. doi: 10.1016/j.ydbio.2018.05.002 . 29753626 PMC6239991

[60] Huber E. Evolution of Facial Musculature and Facial Expression. London, UK: Johns Hopkins Press; 1931.

[61] Kaminski J, Waller BM, Diogo R, Hartstone-Rose A, Burrows AM. Evolution of facial muscle anatomy in dogs. Proc Natl Acad Sci U S A. 2019; 116(29):14677–14681. doi: 10.1073/pnas.1820653116 . 31209036 PMC6642381

[62] Burrows AM, Waller BM, Parr LA. Facial musculature in the rhesus macaque (*Macaca mulatta*): evolutionary and functional contexts with comparisons to chimpanzees and humans. J Anat. 2009; 215(3):320–34. doi: 10.1111/j.1469-7580.2009.01113.x . 19563473 PMC2750044

[63] Burrows AM, Diogo R, Waller BM, Bonar CJ, Liebal K. Evolution of the muscles of facial expression in a monogamous ape: evaluating the relative influences of ecological and phylogenetic factors in hylobatids. Anat Rec (Hoboken). 2011; 294(4):645–63. doi: 10.1002/ar.21355 . 21370494

[64] Burrows AM, Waller BM, Parr LA, Bonar CJ. Muscles of facial expression in the chimpanzee (Pan troglodytes): descriptive, comparative and phylogenetic contexts. J Anat. 2006; 208(2):153–67. doi: 10.1111/j.1469-7580.2006.00523.x . 16441560 PMC2100197

[65] Grammer K, Schiefenhovel W, Schleidt M, Lorenz B, Eibl-Eibesfeldt I. Patterns on the face: the eyebrow flash in crosscultural comparison. Ethology. 1988;77(4):279–299. doi: 10.1111/j.1439-0310.1988.tb00211.x

[66] Schmidt KL, Cohn JF. Human facial expressions as adaptations: Evolutionary questions in facial expression research. Am J Phys Anthropol. 2001; Suppl 33:3–24. doi: 10.1002/ajpa.2001 . 11786989 PMC2238342

[67] Burrows AM. The facial expression musculature in primates and its evolutionary significance. Bioessays. 2008; 30(3):212–225. doi: 10.1002/bies.20719 . 18293360

